# Plasma Volume Score for Early Risk Stratification Following Heart Transplantation: An Exploratory Observational Study

**DOI:** 10.3390/jcm15156011

**Published:** 2026-08-02

**Authors:** Kira Osipenko, Rabab Saleh, Alexandra Kaider, Amila Kahrovic, Arezu Aliabadi-Zuckermann, Daniel Zimpfer, Andreas Zuckermann, Emilio Osorio-Jaramillo

**Affiliations:** 1 Department of Cardiac and Thoracic Aortic Surgery, Medical University of Vienna, 1090 Vienna, Austria; 2 Center for Medical Data Science, Institute of Clinical Biometrics, Medical University of Vienna, 1090 Vienna, Austria

**Keywords:** heart transplantation, plasma volume status, risk stratification

## Abstract

**Background/Objectives**: Plasma volume score (PVS) is a simple integrative surrogate marker derived from body weight and hematocrit that may capture plasma volume expansion, congestion, and overall disease severity. Previous studies have demonstrated its prognostic value in patients with heart failure and other cardiovascular populations. However, the clinical relevance of pre-transplant PVS in heart transplant recipients remains unknown. **Methods**: We retrospectively analyzed 375 adult heart transplant recipients. Baseline PVS was calculated before transplantation and analyzed as both a continuous and categorical variable using a predefined cut-off of 3.1. The primary endpoint was 1-year all-cause mortality. Secondary endpoints included overall survival, 30-day mortality, and post-transplant dialysis. **Results**: Median baseline PVS was −5.4 (interquartile range −12.4 to 2.6), and 88 patients (23.5%) had a PVS ≥ 3.1. In multivariable linear regression analysis, elevated PVS was independently associated with lower estimated glomerular filtration rate, ventricular assist device support, pre-transplant infection, clinical congestion, admission status, bilirubin levels, and body mass index (all *p* < 0.05). Patients with PVS ≥ 3.1 had significantly lower 1-year survival compared with patients with PVS < 3.1 (81.8% vs. 90.9%; log-rank *p* = 0.017). Higher PVS was associated with increased 1-year mortality (hazard ratio [HR] 1.028 per PVS unit, 95% confidence interval [CI] 1.001–1.055; *p* = 0.044). Elevated PVS was additionally associated with post-transplant dialysis (OR 1.14 per 5-point increase, 95% CI 1.01–1.28; *p* = 0.036). No significant association was observed between PVS and overall long-term survival or PGD. **Conclusions**: Elevated pre-transplant PVS is associated with impaired 1-year survival and increased risk of post-transplant dialysis after heart transplantation. As an inexpensive and readily available marker of congestion and disease severity, PVS may provide useful information for pre-transplant risk stratification.

## 1. Introduction

Heart transplantation remains the gold-standard treatment for selected patients with end-stage heart failure. Despite substantial improvements in surgical techniques, immunosuppressive therapy, and perioperative management, early morbidity and mortality after heart transplantation remain clinically relevant. Accurate pre-transplant risk stratification is therefore essential to identify patients at increased risk of adverse postoperative outcomes and to optimize perioperative management.

Congestion is a central feature of advanced heart failure and is strongly associated with disease severity, end-organ dysfunction, and adverse clinical outcomes. However, assessment of volume status remains challenging in routine clinical practice. Plasma volume score (PVS), calculated from body weight and hematocrit, has emerged as a simple surrogate marker of plasma volume expansion and congestion. Because PVS can be derived from routinely available clinical parameters, it represents an attractive tool for risk assessment in patients with advanced cardiovascular disease.

Several studies have demonstrated the prognostic value of PVS in patients with heart failure [1,2,3]. Elevated PVS has been associated with increased mortality and hospitalization in chronic and acute heart failure populations [4]. These findings suggest that PVS reflects not only volume overload but also the overall burden of disease severity and end-organ dysfunction in patients with end-stage heart failure.

However, whether this prognostic information remains relevant after heart transplantation is unknown. In contrast to conventional heart failure populations, heart transplantation replaces the failing heart and corrects the underlying hemodynamic abnormality. It therefore remains unclear whether an elevated pre-transplant PVS merely reflects reversible congestion caused by advanced heart failure or identifies patients with persistent end-organ dysfunction and increased post-transplant risk.

Data regarding the clinical relevance of pre-transplant PVS in heart transplant recipients remain scarce. Therefore, the aim of the present study was to investigate the association between pre-transplant PVS and post-transplant outcomes in heart transplant recipients. Furthermore, we sought to identify clinical factors associated with elevated baseline PVS and to evaluate the potential role of PVS as a tool for pre-transplant risk stratification.

## 2. Materials and Methods

### 2.1. Study Design and Ethical Statement

This monocentric retrospective, explorative study was conducted at the Medical University of Vienna and was approved by the local ethics committee (EK Nr: 1994/2023). Due to the retrospective study design, informed consent was not required.

All patients aged 18 years or older who underwent orthotopic heart transplantation at the Department of Cardiac and Thoracic Aortic Surgery (Vienna General Hospital, Vienna, Austria) between 1 January 2012 and 31 December 2023 were included in the study.

The primary endpoint of the study was 1-year all-cause mortality after heart transplantation. Secondary endpoints included overall survival during follow-up, 30-day mortality, and post-transplant dialysis. In addition, the association of PVS with the development of primary graft dysfunction and factors associated with baseline PVS were evaluated in an exploratory analysis.

For the purposes of this study, preoperative and postoperative patient characteristics, procedural data, laboratory parameters, and follow-up data were collected individually from hospital records. Survival data were obtained from national statistics (Statistics Austria, Vienna, Austria). Patients with missing baseline variables required for PVS calculation or multivariable analyses were excluded from the respective analyses. No imputation of missing data was performed.

### 2.2. Laboratory Data

Laboratory parameters were assessed before heart transplantation (HTX). Preoperative laboratory parameters, defined as baseline, were collected within 7 days prior to surgery.

For the calculation of the plasma volume score (PVS), hematocrit values were obtained via routine blood sampling at the time of patient admission. Additionally, each patient’s weight was measured upon admission to ensure accurate recording of their current body weight.

### 2.3. Plasma Volume Status (PVS) Calculation

Plasma volume status (PVS) was defined as the percentage deviation of estimated actual plasma volume from the ideal value [5]:

PVS (%) = (actual PV − ideal PV)/(ideal PV) × 100

The actual plasma volume (PV) was calculated using hematocrit and body weight with sex-specific constants [6]:

actual PV = (1 − Hct) × [a + (b × weight (kg))]

with a = 1530, b = 41 for males, and a = 864, b = 47.9 for females.

The ideal plasma volume was derived according to Longo et al. [7]:

ideal PV = c × weight (kg)

with c = 39 for males and c = 40 for females.

### 2.4. Statistical Analysis

Continuous variables were summarized as mean ± standard deviation or median with interquartile range, as appropriate. Categorical variables were presented as frequencies and percentages.

Baseline PVS was analyzed both as a continuous variable and as a dichotomous variable using a predefined cut-off value of 3.1, which was selected based on previously published data [8]. In addition, patients were stratified into PVS quartiles for Kaplan–Meier survival analyses.

The Kaplan–Meier method was used to estimate survival probabilities, and the log-rank test was used for comparisons of survival curves. Univariable and multivariate Cox proportional hazards regression models were used to evaluate the potential association of baseline PVS values with 1-year mortality. Binary logistic regression models were used to analyze the association of PVS levels with thirty-day mortality. Multinomial logistic regression models were performed to assess a potential correlation between PVS and the need for dialysis within 30 days, thereby distinguishing between dialysis, early death without dialysis, and 30-day survival without dialysis. The chi-square test and unpaired t-test were used to evaluate a potential association of PVS levels with the development of PGD. Multivariable linear regression analysis was performed to identify determinants of baseline PVS.

A two-sided *p*-value < 0.05 was considered statistically significant. Statistical analyses were performed using SAS version 9.4 (SAS Institute Inc., Cary, NC, USA).

## 3. Results

### 3.1. Study Population

**Figure 1:**
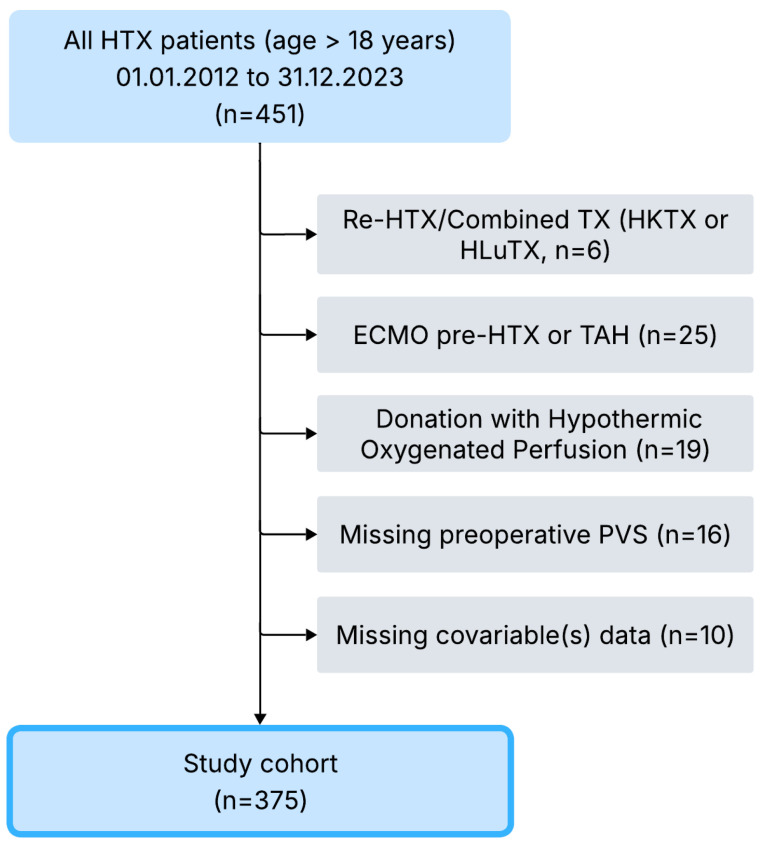
Patient inclusion and exclusion criteria. HKTX, heart–kidney transplantation; HLuTX, heart–lung transplantation; HTX, heart transplantation; POD, postoperative day; PVS, plasma volume score; TAH, total artificial heart; TX, transplantation.

The baseline plasma volume score was available in 375 heart transplant recipients (Figure 1). Baseline characteristics are summarized in Table 1.

The median PVS was −5.4 (interquartile range [IQR] −12.4 to 2.6), with a mean value of −4.9 ± 11.2. Using the predefined cut-off of 3.1, 88 patients (23.5%) were classified as having elevated PVS, whereas 287 patients (76.5%) had a PVS below 3.1.

### 3.2. Overall Survival

During follow-up, 94 deaths occurred. When patients were stratified according to PVS quartiles, estimated 10-year survival rates were 73.2%, 72.3%, 72.4%, and 65.6% across increasing quartiles. However, no significant differences in long-term survival were observed between quartiles (log-rank *p* = 0.147).

Similarly, patients with elevated PVS (≥3.1) showed numerically lower long-term survival than patients with PVS < 3.1 (66.4% vs. 72.3%), although this difference was not statistically significant (log-rank *p* = 0.255).

In univariable Cox regression analysis, baseline PVS as a continuous variable was not associated with overall mortality (hazard ratio [HR] 1.011 per PVS unit, 95% confidence interval [CI] 0.992–1.029, *p* = 0.255). No evidence for a non-linear association was observed (PVS^2^ *p* = 0.843).

### 3.3. One-Year Survival

**Figure 2:**
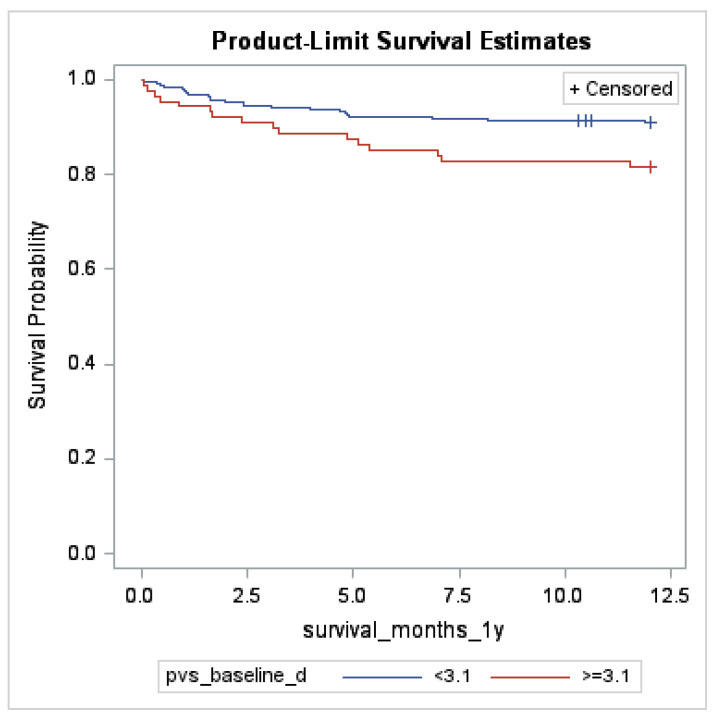
One-year survival according to baseline plasma volume score. Survival stratified according to the predefined PVS cut-off of 3.1. Patients with PVS ≥ 3.1 had significantly lower 1-year survival than patients with PVS < 3.1 (log-rank *p* = 0.017).

Within the first year after transplantation, 42 deaths occurred. Using the predefined cut-off of 3.1, patients with elevated PVS had significantly lower 1-year survival compared with patients with PVS < 3.1 (Figure 2, 81.8% vs. 90.9%; log-rank *p* = 0.017).

In univariable Cox regression analysis, higher baseline PVS was associated with increased risk of death within the first year after transplantation (HR 1.028 per PVS unit, 95% CI 1.001–1.055, *p* = 0.044). For every 5-point increase in PVS, the hazard of 1-year mortality increased by 14.6% (HR 1.146, 95% CI 1.004–1.308).

When PVS was analyzed as a dichotomous variable, patients with PVS ≥ 3.1 had more than twice the risk of 1-year mortality compared with patients with PVS < 3.1 (HR 2.10, 95% CI 1.13–3.91, *p* = 0.020).

In a multivariable Cox regression model (Table 2A,B) adjusting for recipient age, cold ischemic time, IMPACT score, and donor–recipient sex mismatch, baseline PVS remained independently associated with 1-year mortality (HR 1.029 per PVS unit, 95% CI 1.001–1.058, *p* = 0.040). Recipient age was also independently associated with mortality (HR 1.039 per year, 95% CI 1.003–1.077, *p* = 0.036). Expressed per 5-unit increase, the adjusted HR for PVS was 1.156 (95% CI 1.007–1.328).

Likewise, in the adjusted model using the predefined cut-off (Table 2A,B), PVS ≥ 3.1 remained an independent predictor of 1-year mortality (HR 2.11, 95% CI 1.11–4.02, *p* = 0.024).

### 3.4. Early Post-Transplant Outcomes

#### 3.4.1. Thirty-Day Mortality

Thirty-day mortality occurred in 11 patients (2.9%). Baseline PVS showed a borderline association with 30-day mortality when analyzed as a continuous variable (odds ratio [OR] 1.05 per PVS unit, 95% CI 0.998–1.106, *p* = 0.061). For every 5-point increase in PVS, the OR for 30-day mortality was 1.28 (95% CI 0.99–1.66).

Patients with PVS ≥ 3.1 had a numerically increased risk of 30-day mortality compared with patients with PVS < 3.1 (OR 2.82, 95% CI 0.84–9.48), although this difference did not reach statistical significance (*p* = 0.093).

#### 3.4.2. Post-Transplant Dialysis

Among the 375 recipients, 67 patients (17.9%) required dialysis after transplantation and nine patients (2.4%) died before discharge without preceding dialysis.

In multinomial logistic regression analysis, higher baseline PVS was associated with an increased risk of post-transplant dialysis (OR 1.14 per 5-point increase, 95% CI 1.01–1.28, *p* = 0.036). When using the predefined cut-off of 3.1, no significant association with post-transplant dialysis was observed.

#### 3.4.3. Primary Graft Dysfunction

Primary graft dysfunction (PGD) occurred in 47 patients (12.5%). Mean baseline PVS was numerically higher among patients who developed PGD than among those without PGD (−2.8 ± 12.1 vs. −5.2 ± 11.1), but the difference was not statistically significant (*p* = 0.167). Likewise, the incidence of PGD did not differ between patients with PVS < 3.1 and those with PVS ≥ 3.1 (11.9% vs. 14.8%, *p* = 0.468).

### 3.5. Determinants of Baseline PVS

In univariable linear regression analyses, higher PVS values were associated with lower body mass index (BMI; β = −1.26, *p* < 0.001), impaired renal function (log-transformed eGFR; β = −2.84, *p* = 0.002), dialysis prior to transplantation (β = 9.80, *p* < 0.001), pre-transplant infection (β = 8.66, *p* < 0.001), catecholamine support (β = 10.22, *p* < 0.001), and congenital/other cardiomyopathy (β = 5.03, *p* = 0.014). A non-linear association between BMI and PVS was observed (BMI^2^ *p* = 0.007).

In multivariable linear regression analysis (Table 3), baseline PVS remained independently associated with BMI (*p* < 0.0001), reduced renal function (log-transformed eGFR: β = −3.04, *p* = 0.001), VAD support (β = 4.54, *p* < 0.001), pre-transplant infection (β = 3.26, *p* = 0.029), bilirubin levels (log2-transformed, β = −1.40, *p* = 0.003), clinical congestion (β = 1.50, *p* = 0.018), and admission status before transplantation (overall *p* = 0.0007). Compared with patients admitted to the intensive care unit, patients admitted from a regular ward (β = −5.26, *p* = 0.010) or from home (β = −7.38, *p* < 0.001) had significantly lower PVS values. The final model explained 43.0% of the variability in baseline PVS (R^2^ = 0.43).

## 4. Discussion

In this single-center cohort of adult heart transplant recipients, elevated pre-transplant PVS was associated with impaired early outcome after heart transplantation. The main finding was that patients with PVS ≥ 3.1 had significantly lower 1-year survival than patients with PVS < 3.1. In contrast, PVS was not significantly associated with overall long-term survival. These results suggest that PVS may primarily identify patients with increased early post-transplant vulnerability rather than patients with a generally worse long-term prognosis.

PVS is a simple marker derived from body weight and hematocrit and is intended to reflect the deviation of estimated plasma volume from the ideal plasma volume. In our cohort, higher PVS was independently associated with markers of advanced pre-transplant disease severity, including impaired renal function, VAD support, pre-transplant infection, clinical congestion, and admission status before transplantation. Patients treated in the intensive care unit had higher PVS values than patients admitted from a regular ward or from home. These findings suggest that PVS may not represent plasma volume alone but rather an integrative marker of recipient vulnerability, incorporating information related to renal dysfunction, hemodilution, inflammation, body composition, congestion, and overall disease severity.

Previous studies have demonstrated the prognostic relevance of estimated plasma volume status in patients with heart failure. Grodin et al. reported that elevated plasma volume status was independently associated with adverse long-term outcomes in patients with heart failure with preserved ejection fraction [4]. Similarly, Soetjoadi et al. demonstrated an association between elevated estimated plasma volume status and mortality in patients with right-sided heart failure [9]. Collectively, these studies suggest that plasma volume expansion reflects a higher-risk heart failure phenotype characterized by congestion and end-organ dysfunction.

Beyond heart failure populations, the prognostic relevance of plasma volume-derived indices has been demonstrated in several cardiovascular populations. Schaefer et al. extended the concept of plasma volume status to a cardiac surgical population and demonstrated that elevated PVS was associated with adverse postoperative outcomes in patients undergoing mitral valve surgery [8]. Importantly, the PVS threshold of 3.1 applied in the present study was derived from this work, allowing us to evaluate its prognostic relevance in a heart transplant population. Similarly, Hasimbegovic et al. demonstrated that deviations from ideal plasma volume were associated with increased mortality following isolated tricuspid valve surgery [10].

The prognostic relevance of PVS has also been demonstrated in a variety of high-risk clinical settings. Elevated PVS has been associated with adverse outcomes following LVAD implantation [11], coronary artery bypass grafting [12], and transcatheter aortic valve replacement [13,14], and has even been linked to increased mortality in critically ill patients with acute respiratory distress syndrome [15]. Collectively, these findings support the concept that plasma volume expansion reflects a vulnerable clinical phenotype characterized by congestion, end-organ dysfunction, and increased risk of adverse outcomes across different disease states.

However, heart transplantation differs fundamentally from conventional heart failure populations because the failing heart is replaced. This may explain why PVS predicted early mortality in our cohort but was not significantly associated with long-term survival. Elevated PVS may identify patients who enter transplantation in a more vulnerable physiological state. However, once patients survive the perioperative and early post-transplant period, long-term outcomes are increasingly determined by graft-related factors rather than the recipient’s pre-transplant condition. In patients with advanced heart failure, elevated PVS reflects ongoing cardiac dysfunction and persistent congestion. In contrast, successful heart transplantation corrects the underlying hemodynamic abnormality. Consequently, the prognostic impact of elevated pre-transplant PVS may be strongest during the perioperative and early postoperative period, whereas long-term outcome becomes increasingly determined by graft-related factors such as rejection, infection, malignancy, chronic kidney disease, and cardiac allograft vasculopathy.

The association between PVS and post-transplant dialysis is also clinically relevant. Higher PVS was associated with an increased risk of dialysis after transplantation when analyzed as a continuous variable. This finding is consistent with the independent association between baseline PVS and reduced pre-transplant renal function. Patients with elevated PVS had significantly impaired renal function before transplantation, suggesting that PVS may capture reduced renal reserve. Consequently, these patients may be more susceptible to perioperative kidney injury and the hemodynamic stress associated with transplantation.

The absence of an association with primary graft dysfunction is noteworthy. PGD is primarily determined by donor-, procedural-, and graft-related factors. In contrast, PVS was associated with outcomes that are more closely linked to recipient characteristics, particularly early mortality and post-transplant dialysis. This observation supports the hypothesis that PVS captures recipient vulnerability rather than susceptibility to graft-related injury.

The recent literature also highlights limitations of estimated plasma volume markers. Ahlgrim et al. showed that estimated PVS has only modest accuracy for detecting true plasma volume excess in compensated chronic heart failure [16]. Therefore, PVS should not be interpreted as a direct replacement for invasive or quantitative volume assessment. Rather, it should be considered an easily available surrogate marker that integrates information from volume status, hemodilution, body composition, and disease severity. Similarly, serum albumin has been shown to provide complementary prognostic information in patients with heart failure, reflecting inflammation, nutritional status, hepatic congestion, renal dysfunction, and impaired oncotic pressure. Therefore, future studies should evaluate whether combining PVS with complementary biomarkers and established transplant-specific risk models, such as the IMPACT score, further improves individualized pre-transplant risk stratification [17,18].

From a clinical perspective, PVS possesses several attractive characteristics. It can be calculated easily from routinely available variables, requires no additional laboratory testing, imaging, or invasive assessment, and is available in virtually every heart transplant candidate at no additional cost. Given its association with impaired 1-year survival and post-transplant dialysis, PVS may serve as a practical tool for pre-transplant risk stratification. These findings may support future studies investigating the optimization of volume status, closer perioperative monitoring, and the implementation of renal-protective strategies. Although PVS should not be considered a direct measurement of plasma volume, it appears to provide a simple integrative marker of congestion, end-organ dysfunction, and overall disease severity. Future studies should evaluate whether the incorporation of PVS into existing transplant risk models improves prediction of early post-transplant outcomes and whether the targeted reduction of elevated PVS before transplantation can translate into improved clinical outcomes.

This study has several limitations. First, it was a retrospective single-center analysis, and residual confounding cannot be excluded. Furthermore, changes in perioperative management and immunosuppressive protocols during the long inclusion period may have influenced post-transplant outcomes. In addition, the relatively limited number of one-year mortality events may have reduced statistical power and increased the risk of model overfitting despite adjustment for clinically relevant covariates. Therefore, the findings should be interpreted with caution and require validation in larger multicenter cohorts. Second, the predefined PVS cut-off of 3.1 was adopted from a mitral valve surgery population and has not been specifically validated in heart transplant recipients. Therefore, it should not be considered a definitive clinical decision threshold and requires validation in independent multicenter heart transplant cohorts.

## 5. Conclusions

In conclusion, elevated pre-transplant PVS may identify heart transplant recipients at increased risk of impaired 1-year survival and post-transplant dialysis. PVS appears to integrate information related to congestion, renal dysfunction, and overall recipient vulnerability before transplantation. As a simple, inexpensive, and routinely available marker, PVS may improve early risk stratification in heart transplant candidates and may help identify patients requiring intensified perioperative management. Further multicenter studies are needed to validate these findings and to determine whether optimization of pre-transplant volume status can improve early post-transplant outcomes.

## Figures and Tables

**Table 1:** Baseline characteristics. Bold values indicate statistically significant differences between groups (*p* < 0.05).

Variables	All Patients (*n* = 375)	PVS < 3.1 (*n* = 287) Median (IQR)	PVS ≥ 3.1 (*n* = 88) Median (IQR)	*p*-Value
Age (years), 25th-75th interval	58 (50–64)	58 (51–64)	58 (46–64)	0.689
Gender male, *n* (%)	93 (24.8%)	68 (23.7)	25 (28.4)	0.370
Body mass index, kg/m^2^	26 (23–29)	26.6 (24.0–29.0)	24.0 (21.6–27.0)	**<0.001**
Weight (VAD-adjusted), kg	79 (70–89)	81.0 (72.0–91.3)	72.0 (61.4–81.7)	**<0.001**
Height, cm	175 (168–181)	177.0 (169.0–182.0)	172.5 (167.0–180.0)	**0.004**
Diabetes mellitus (pre-HTX), *n* (%)	99 (26.4%)	82 (28.6%)	17 (19.3%)	0.085
Chronic kidney disease (pre-HTX), *n* (%)	147 (39.2%)	104 (36.2)	43 (48.9)	**0.034**
Dialysis (pre-HTX)	15 (4.0%)	7 (2.4%)	8 (9.1%)	0.010 *
GFR (pre-HTX), ml/min	68 (50–90)	69.3 (51.7–91.5)	61.6 (42.8–87.1)	0.075
History of stroke (pre-HTX), *n* (%)	25 (6.7%)	21 (7.3)	4 (4.5)	0.362
Previous surgery, *n* (%)	165 (44.0%)	121 (42.2)	44 (50.0)	0.195
VAD in place, *n* (%)	113 (30.1%)	90 (31.4)	23 (26.1)	0.350
Infection (pre-HTX), *n* (%)	52 (13.9%)	27 (9.4)	25 (28.4)	**<0.001**
Ischemic cardiomyopathy, *n* (%)	128 (34.1%)	97 (33.8)	31 (35.2)	0.805
Non-ischemic cardiomyopathy, *n* (%)	208 (55.5%)	165 (57.5)	43 (48.9)	0.154
Congenital cardiomyopathy, *n* (%)	3 (0.8%)	3 (1.0)	0 (0.0)	1.000 *
Another cardiomyopathy, *n* (%)	36 (9.6%)	22 (7.7)	14 (15.9)	**0.022**

Continuous variables are presented as median (interquartile range [IQR]) and were compared using the Mann–Whitney U test. Categorical variables are presented as *n* (%) and were compared using Pearson’s χ^2^ test or Fisher’s exact test, as appropriate. PVS groups were defined using the predefined cut-off value of 3.1. * *p*-value derived from Fisher’s exact test. GFR, glomerular filtration rate; HTX, heart transplantation; IQR, interquartile range; PVS, plasma volume status; VAD, ventricular assist device.

**Table 2:** Association of baseline plasma volume score with 1-year mortality after heart transplantation.

(**A**)**. Continuous PVS**		
**Variables**	**Multivariable** **HR (95% CI)**	***p*** **-Value**
PVS (per 1-unit increase) *	1.029 (1.001–1.058)	**0.040**
Recipient age (per year)	1.039 (1.003–1.077)	**0.036**
Cold ischemic time (per minute)	1.003 (0.998–1.009)	0.209
IMPACT score	0.977 (0.892–1.070)	0.617
Donor–recipient sex mismatch	0.876 (0.388–1.979)	0.750
(**B**)**. Dichotomized PVS**		
**Variables**	**Multivariable** **HR (95% CI)**	***p*** **-Value**
PVS ≥ 3.1 vs. <3.1	2.107 (1.106–4.015)	**0.024**
Recipient age (per year)	1.038 (1.001–1.075)	**0.042**
Cold ischemic time (per minute)	1.003 (0.998–1.009)	0.222
IMPACT score	0.984 (0.901–1.075)	0.722
Donor–recipient sex mismatch	0.880 (0.390–1.986)	0.758

Values are presented as hazard ratios (HRs) with 95% confidence intervals (CIs). Hazard ratios were derived from multivariable Cox proportional hazards regression models adjusted for recipient age, cold ischemic time, IMPACT score, and donor–recipient sex mismatch. In Panel A, PVS was analyzed as a continuous variable. In Panel B, PVS was analyzed as a dichotomous variable using the predefined cut-off value of 3.1. CI, confidence interval; HR, hazard ratio; IMPACT, index for mortality prediction after cardiac transplantation; PVS, plasma volume score. * Hazard ratio for PVS per 5-unit increase: HR 1.156 (95% CI 1.007–1.328).

**Table 3:** Multivariable linear regression analysis of factors associated with baseline plasma volume score (PVS). Bold values indicate statistically significant differences between groups (*p* < 0.05)

Variables	Multivariable β (SE)	*p*-Value
Age (per year)	−0.10 (0.05)	0.055
Sex	−0.34 (1.14)	0.769
BMI *	-	**<0.0001**
Cardiomyopathy type ^†^	-	0.276
eGFR (log2)	−3.04 (0.93)	**0.001**
Dialysis before HTX	2.72 (2.60)	0.297
VAD support	4.54 (1.07)	**<0.001**
Pre-transplant infection	3.26 (1.49)	**0.029**
Bilirubin (log2)	−1.40 (0.46)	**0.003**
Catecholamine support	2.37 (2.08)	0.255
Clinical congestion score	1.50 (0.63)	**0.018**
NYHA class	0.88 (0.67)	0.188
Admission status ^‡^	-	**0.0007**
Admission from ward vs. ICU	−5.26 (2.03)	**0.010**
Admission from home vs. ICU	−7.38 (1.93)	**<0.001**

Values are presented as regression coefficients (β) derived from a multivariable linear regression model for baseline plasma volume score (PVS). Body mass index (BMI) was modeled using a quadratic term (BMI and BMI^2^) to account for the observed nonlinear relationship with PVS. Admission status was entered as a categorical variable with intensive care unit (ICU) admission as the reference category. The final model explained 43.0% of the variability in baseline PVS (R^2^ = 0.43). * BMI was modeled using a quadratic term (BMI and BMI^2^); the reported *p* value represents the overall test for BMI. † cardiomyopathy type was entered as a categorical variable with ischemic cardiomyopathy as the reference category; the reported *p* value represents the overall test for cardiomyopathy type. ‡ admission status was entered as a categorical variable with ICU admission as the reference category; the reported *p* value represents the overall test for admission status. BMI, body mass index; eGFR, estimated glomerular filtration rate; HTX, heart transplantation; ICU, intensive care unit; NYHA, New York Heart Association; PVS, plasma volume score; SE, standard error; VAD, ventricular assist device.

## Data Availability

The datasets presented in this article are not readily available due to patient privacy and ethical restrictions.
